# Targeting efflux pumps prevents the multi-step evolution of high-level resistance to fluoroquinolone in *Pseudomonas aeruginosa*

**DOI:** 10.1128/spectrum.02981-24

**Published:** 2025-02-21

**Authors:** Xiao-Quan Yu, Hao Yang, Han-Zhong Feng, Jun Hou, Jun-Qiang Tian, Shao-Min Niu, Chong-Ge You, Xuan-Yu Tao, Si-Ping Zhang, Zhi-Ping Wang, Yong-Xing He

**Affiliations:** 1 Institute of Urology, Gansu Province Clinical Research Center for urinary system disease, The Second Hospital & Clinical Medical School, Lanzhou University, Lanzhou, Gansu, People's Republic of China; 2 Ministry of Education Key Laboratory of Cell Activities and Stress Adaptations, School of Life Sciences, Lanzhou University, Lanzhou, Gansu, People's Republic of China; 3 Laboratory Medicine Center, The Second Hospital of Lanzhou University, Lanzhou, Gansu, People's Republic of China; 4 Institute for Environmental Genomics, University of Oklahoma, Norman, Oklahoma, USA; Ocean University of China, Qingdao, China

**Keywords:** antimicrobial resistance evolution, efflux pump, *Pseudomonas aeruginosa*

## Abstract

Antibiotic resistance is emerging as a significant global health crisis, necessitating the urgent development of novel antibiotics or alternative therapies. Although it is recognized that bacteria require multiple mutations to develop resistance levels exceeding the mutant prevention concentration, the specific mutation combinations conferring high resistance have been largely undefined. Here, we investigated the multi-step evolution of fluoroquinolone resistance in *Pseudomonas aeruginosa* through experimental evolution and whole-genome sequencing coupled with proteomic approaches. We discovered that in low-dose and high-dose experimental evolution scenarios, combinations of mutations in the negative regulators of efflux pumps (*nfxB/mexR*) and DNA gyrases (*gyrA/gyrB*) contributed to the high-level resistance and some of these combinations were also prevalent in clinical isolates of *P. aeruginosa*. Notably, the selected *nfxB* mutation, which resulted in the overexpression of the MexCD-OprJ efflux pump, also exhibited collateral sensitivity to aminoglycosides and enhanced antibiotic tolerance. It was further revealed that the efflux pump inhibitor phenylalanine-arginine β-naphthylamide (PAβN) could effectively prevent evolution to high-level resistance for both laboratory and clinical *P. aeruginosa* strains. Our work highlights the critical role of efflux pump repressor-related mutations in the evolution of high-level antibiotic resistance and demonstrates the potential of targeting these mutations to impede the evolution toward high-level resistance.

**IMPORTANCE:**

In this study, we examined the stepwise evolution of fluoroquinolone resistance in *Pseudomonas aeruginosa* using experimental evolution, whole-genome sequencing, and proteomic analyses. Our findings revealed that under both low-dose and high-dose conditions, mutations in efflux pump regulators (*nfxB*/*mexR*) and DNA gyrase genes (*gyrA*/*gyrB*) synergistically contributed to high-level resistance. These mutation combinations were not only observed in experimental settings but also detected in clinical isolates of *P. aeruginosa*. This work underscores the pivotal role of efflux pump repressor-related mutations in the progression to high-level antibiotic resistance. It also highlights the promise of targeting efflux pumps as a strategy to prevent the multi-step evolution of resistance in *P. aeruginosa*.

## INTRODUCTION

The excessive use of antibiotics has resulted in significant problems related to antimicrobial resistance, posing great challenges to treating clinical infections (1). The absence of relevant countermeasures may lead to drug-resistant infections endangering 10 million lives and causing an economic output loss worth 100 trillion USD annually by 2050 (2). Resistance is perhaps the most common strategy for bacteria to survive antibiotics, allowing bacteria to proliferate continuously in the presence of high levels of antibiotics. This can be acquired through horizontal gene transfer and/or spontaneous chromosomal mutations (3, 4), which can help bacteria survive antibiotics through antibiotic inactivation, drug target modification, and increased antibiotic efflux (5–7). To solve this global challenge, there is an urgent need to develop novel antibiotics for treating drug-resistant infections and new approaches to prevent the evolution and dissemination of antibiotic resistance in pathogens (8).

Antibiotic resistance is most commonly measured by the minimum inhibitory concentration (MIC), which is the lowest concentration of the antibiotic required to prevent bacterial growth (9). However, when exposing a large number of bacterial cells (e.g., an inoculum of 10^10^ cells) to antibiotics at MIC levels, there will typically be a sub-population of pre-existing single-step resistant mutants due to spontaneous mutations. Even when the antibiotic concentration surpasses the MIC, these single-step mutants will persist until a concentration threshold is reached, which is defined as the mutant prevention concentration (MPC) (10). Above this threshold, multi-step resistant mutations are required for the bacterial population to survive. Mathematical modeling has demonstrated that if more than two mutations are required to survive the antibiotics, resistance evolution will be substantially restricted, and the extent of this limitation depends on the combination of drug type and pharmacokinetic properties (11). However, achieving and maintaining such concentrations can be quite challenging, owing to the pharmacokinetics and toxicity of antibiotics. Moreover, high-level resistance conferred by multi-step mutations can emerge even during low-level antibiotic exposure (12). Therefore, how to prevent the occurrence and propagation of multi-step, high-resistance mutants is essential in treating and managing drug-resistant pathogens.

Within an infected host, the resistance evolution of pathogens is mainly driven by chromosomal mutations rather than horizontal gene transfer (13, 14). Single-step chromosomal mutations may confer some level of resistance, but in most cases, they are not sufficient to push the MIC of the evolved clones significantly above the MPC of the ancestral strain (11, 15). Higher-level resistance, such as a MIC significantly exceeding the MPC, typically requires multi-step mutations that collectively provide a more substantial defense against the antibiotic’s effects (16). Combinations of mutations conferring high-level resistance (with MIC exceeding the ancestral MPC) were generally thought to be mainly dependent on the types of antibiotics and genetic background of bacterial strains. Chloramphenicol and doxycycline resistance in *Escherichia coli* emerges from mutations affecting translation, transcription, and transport, but trimethoprim resistance results solely from gene alterations in the dihydrofolate reductase enzyme (17). Therefore, understanding the mechanism of the multi-step evolution toward high-level resistance provides important insights into the mechanisms that restrict the evolutionary escape of drug-resistance pathogens, which may guide the design of effective and sustainable antibiotic therapy (18).

Fluoroquinolone antibiotics are widely used to treat infections caused by *Pseudomonas aeruginosa*. However, their effectiveness is diminishing due to the rapid emergence of bacterial resistance, which is often characterized by the overproduction of drug efflux pumps and alterations in drug targets (19–21). Among drug efflux pumps, overproduction of the MexAB-OprM and MexCD-OprJ efflux pumps has been shown to contribute to high-level fluoroquinolone resistance in both clinical isolates and laboratory experiments (16, 22–24). More recently, the role of MexEF-OprN in low-level fluoroquinolone resistance has gained attention, although it is rarely detected in clinical isolates for impaired virulence (25–27). These observations underscore the potential of targeting efflux pumps as a strategy to prevent the evolution of antimicrobial resistance.

In this work, we used experimental evolution to investigate the multi-step evolution mechanism of fluoroquinolone resistance in *Pseudomonas aeruginosa*, an important opportunistic pathogen that typically causes long-lasting infections in immunocompromised patients. Whole-genome sequencing revealed that exposing bacteria to high-dose (above the MPC of ancestral strain) fleroxacin rapidly selected highly resistant multi-step mutations within approximately 70 generations with a 100% prevalence in the evolved populations (seven treatment cycles), whereas at low-dose (below the MIC of ancestral strain) fleroxacin, multi-step high-resistant mutants only occurred at a much lower rate of ~3 × 10^−5^ in the evolved populations (three treatment cycles). In both experimental evolution scenarios, combinations of mutations in the DNA gyrases (*gyrA/gyrB*) and negative regulators of efflux pumps (*nfxB/mexR*) contributed to the high-level resistance and some of these combinations were also prevalent in clinical isolates of *P. aeruginosa*. Accordingly, we discovered that the efflux inhibitor phenylalanine-arginine β-naphthylamide (PAβN) could effectively prevent the evolution to high-level resistance for both laboratory and clinical *P. aeruginosa* strains. Moreover, the emergence of parallel mutations in *nfxB*, which enhance expression of the MexCD-OprJ efflux pump, also exhibited collateral sensitivity to aminoglycosides and significantly enhanced antibiotic tolerance. Our findings highlight the significant role of efflux pump-related mutations in the multi-step evolution of high resistance and provide proof-of-concept evidence for impeding the evolution toward high-level resistance by targeting the antibiotic efflux pumps.

## RESULTS

### The resistance profiles of bacterial populations selected at sub-MIC and >MPC doses differ

**Fig 1:**
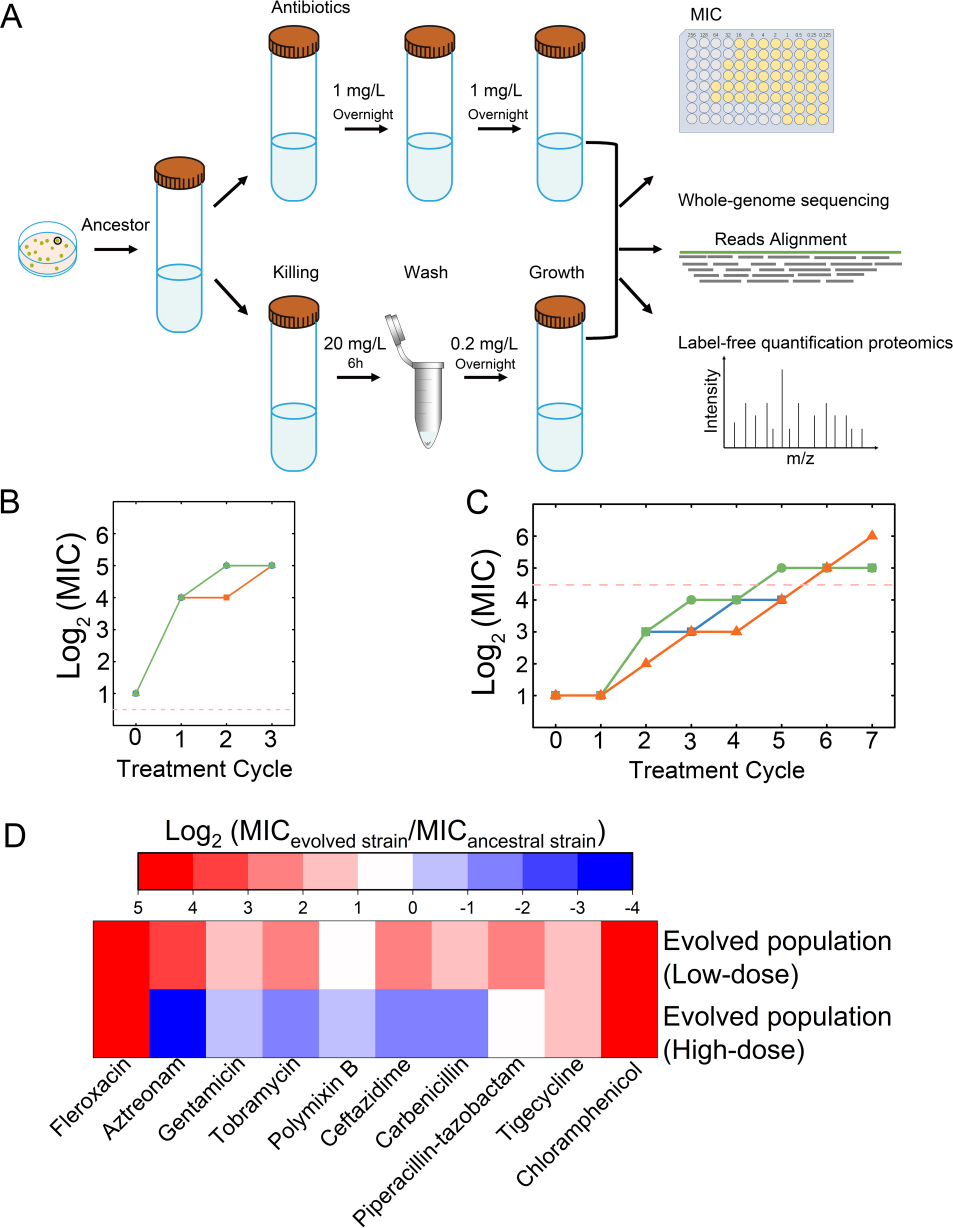
The evolutionary outcomes of resistant bacterial populations differ between different exposure modes. (**A**) Experimental design for (i) low-dose exposure mode: in each cycle, a small-volume overnight culture was resuspended in a larger volume of fresh medium containing 1 mg/L fleroxacin for 24 h and (ii) high-dose exposure mode: in each cycle, a small-volume overnight culture was resuspended in a larger volume of fresh medium containing 20 mg/L fleroxacin for 6 h. After the antibiotic was washed out, the culture was resuspended in a fresh medium containing 0.2 mg/L fleroxacin and grown overnight. (**B**) MIC assay in evolved strains from low-dose exposure mode. Drug concentration (20 mg/L) used in exposure mode was marked as a light red dashed line. (**C**) MIC assay in evolved strains from high-dose exposure mode. Drug concentration (1 mg/L) used in exposure mode was marked as a light red dashed line. (**D**) Multidrug resistance assay in fleroxacin-resistant strains evolved from high-dose exposure mode and low-dose exposure mode. Relative MIC to wild-type *P. aeruginosa* PAO1 strain was measured and colored from blue to red.

To investigate the multi-step resistance evolution in *Pseudomonas aeruginosa*, we conducted laboratory evolution experiments using *P. aeruginosa* PAO1 strain, exposing it to fleroxacin, a fluoroquinolone antibiotic targeting DNA gyrase in Gram-negative bacteria (Fig. S1A) (28). The experiment was designed around two regimens: a continuous low-dose exposure (1 mg/L, below the MIC of 2 mg/L) and an intermittent high-dose exposure (20 mg/L, surpassing the MPC of 9.6 mg/L). In the low-dose regimen, daily dilutions were conducted in a medium containing 1 mg/L fleroxacin (Fig. 1A). For the high-dose regimen, cultures underwent 6 h treatments with 20 mg/L fleroxacin, followed by overnight incubation in a medium with a residual fleroxacin concentration (0.2 mg/L), before being diluted in the same high-dose medium (Fig. 1A). This cycle was repeated for both regimens, each having three replicates, with daily monitoring of the population-level MICs.

Interestingly, under the low-dose regimen, rapid onset of resistance was observed, with all replicate cultures reaching a MIC of 16 mg/L after the first cycle of antibiotic treatment, escalating to 32 mg/L by the end of the third cycle of antibiotic treatment (Fig. 1B). In contrast, under the high-dose regimen, resistance development was gradual, with all replicates reaching a MIC of 32 mg/L after six treatment cycles (Fig. 1C). Extending our resistance analysis to seven additional classes of antibiotics affecting diverse targets, we observed a general pattern of resistance enhancement in the low-dose selected lineages, with at least a twofold increase in resistance across all tested antibiotics, except for polymyxin B. In contrast, in the high-dose selected populations, we noted a decreased resistance to six antibiotics, including gentamicin, tobramycin, aztreonam, carbenicillin, polymyxin B, and ceftazidime. These distinct resistance patterns suggested that while low-dose exposure fostered a broad-spectrum antibiotic resistance in *P. aeruginosa*, high-dose exposure led to collateral sensitivity toward certain antibiotic classes, as a trade-off in the evolution of resistance. These results indicate a fundamental divergence in genetic adaptation mechanisms under different antibiotic selective pressures (Fig. 1D) (29).

### High-dose fleroxacin selects multi-step mutations conferring high resistance toward fluoroquinolone

**Fig 2:**
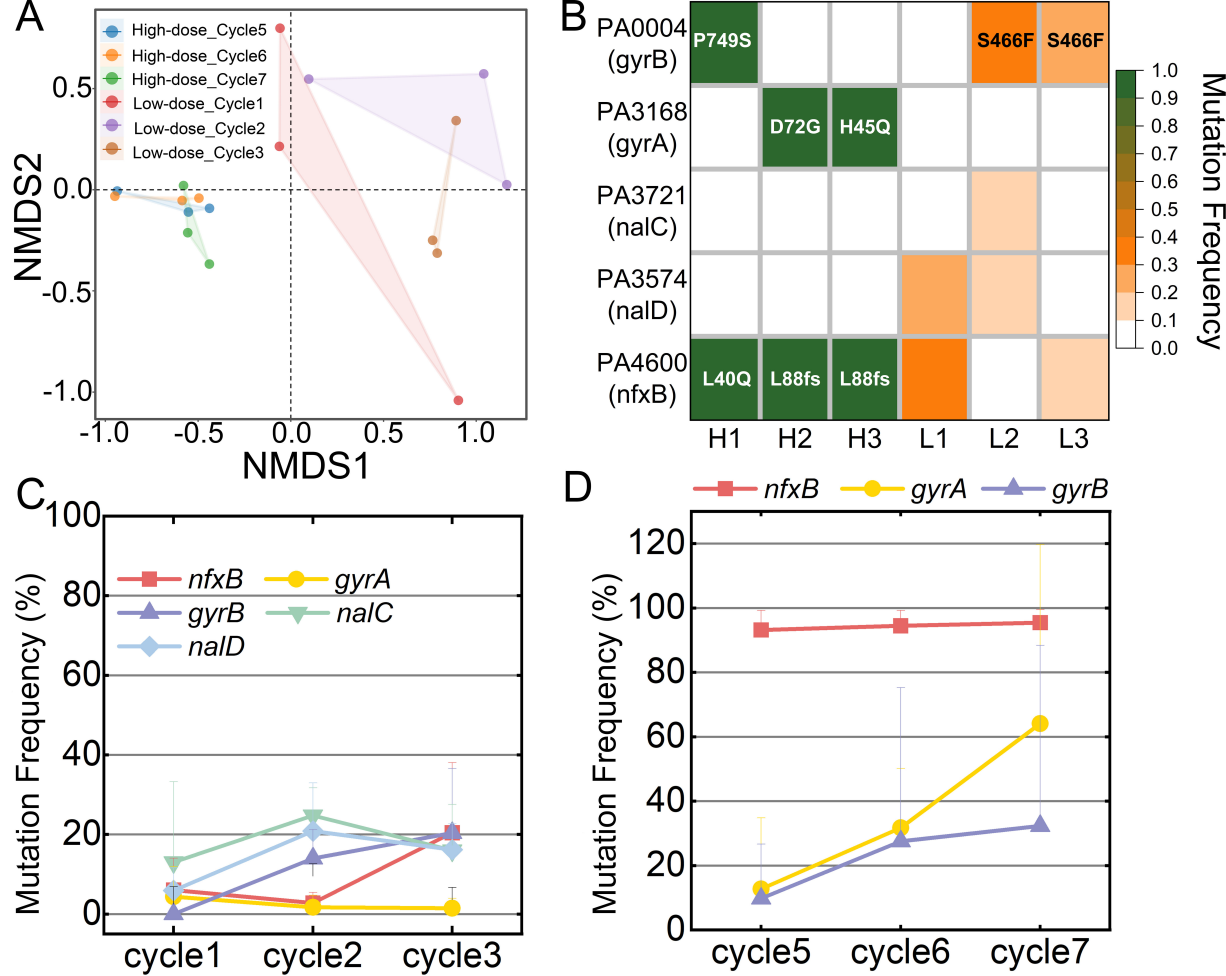
Whole-genome sequencing detected fluoroquinolone-resistance-related mutations with mutation frequency >10% in resistance strains evolved from different exposure modes. (**A**) NMDS plot of population mutation profiles at the gene level (nonsynonymous mutations) based on the Bray-Curtis dissimilarity for all mutations acquired for each population. (**B**) Summary of genes with mutation frequency >10% (rows) that acquired mutations in resistant populations (columns). HI, H2, and H3 denoted three resistant populations evolved from high-dose exposure mode, while L1, L2, and L3 denoted low-dose evolved populations. Boxes are colored based on the frequency of the mutation. Specific mutations of *nfxB*, *gyrA,* and *gyrB* were shown. (**C**) Mutational frequency of key genes in different cycles in the low-dose exposure mode. (**D**) Mutational frequency of key genes in different cycles in the high-dose exposure mode. Three independent experiments were performed and the error bars were calculated.

To identify the mutations responsible for the adaptation under the low-dose or high-dose regimens, the replicates at different cycles of antibiotic treatments were whole-genome sequenced, and their sequences were compared to that of the ancestral PAO1 strain to identify potential point mutations and structural alterations (deletions, duplications, insertions, and inversions). We monitored the mutated genes that reached a frequency of at least 10% since these were likely to be under positive selection given the large population size (∼10^11^ cells at transfer) (30, 31). Bray-Curtis dissimilatory data were calculated at the gene level to evaluate the overall genetic similarity between the evolved populations (32). The differences between the evolved populations from the different treatment regimens and cycles can be viewed in a nonmetric multidimensional scaling (NMDS) plot (Fig. 2A) (33, 34). Considering the total mutational profiles, it was clear that there were significant genetic differences between low-dose and high-dose treated groups and populations were more genetically similar within the high-dose treated group than those within the low-dose treated group (*P* value = 0.001, Fig. S2). In quantifying the genomic alterations, the low-dose and high-dose treatment groups in the final cycle exhibited 40 and 52 mutated genes, respectively (Tables S1 and S2). Among these, 21 mutated genes were common in both groups, with *nfxB*, *gyrA,* and *gyrB* being established markers associated with fluoroquinolone antibiotic resistance (35). Specifically, the low-dose group harbored mutations in *nalD* and *nalC*, all of which are implicated in the regulation of the MexAB-OprM efflux pump, which is involved in transporting a wide range of antibiotics such as β-lactams, fluoroquinolones, and macrolides (36, 37). In contrast, the high-dose population displayed no more unique known mutations linked to resistance (Fig. 2B).

Temporal analysis revealed a progressive accumulation of these mutations, providing insight into the development of the observed multidrug-resistant phenotype in populations that evolved from low-dose or high-dose exposure mode. Mutations related to antibiotic resistance with frequencies >10% of each cycle in low-dose regimen were monitored, in which mutation frequencies of *nalC*, *nalD*, *nfxB*, *gyrB* were gradually increased and finally reached ~20%, indicating that low dose-induced resistant populations exhibited diverse genotypic profiles (Fig. 2C). We next checked into the whole-genome sequencing from cycle 5 to 7 in the high-dose regimen, revealing multiple mutations with frequencies over 90%. In cycle 5, which corresponds to a MIC of 16 or 32 mg/L, all three replicates showed high-frequency (above 90%) mutations in *nfxB*, *pilW*, and *kinB*, consisting of either missense or frameshift mutations. By cycle 7, corresponding to a MIC of 32 or 64 mg/L, additional high-frequency (above 92%) missense mutations emerged, specifically in *gyrA* (D72G or H45Q) and *gyrB* (P749S) (Tables S1 and S2). Although the mutation of *gyrA* (D72G) is not within the classical quinolone resistance-determining regions, it is in the vicinity of the classical quinolone resistance-determining region. The H45Q mutation in *gyrA*, which was first identified in this study, contributes to high-level resistance and could represent a potential resistance target for fluoroquinolones. The P749S mutation of *gyrB* has also not been reported to be related to fluoroquinolone resistance. A time series analysis indicated that these *gyrA* and *gyrB* mutations developed in a time-dependent manner (Fig. 2D). Given that the MIC in cycle 7 was twice that of cycle 5, the combination of *gyrA* (D72G or H45Q) or *gyrB* (P749S) mutations, in the presence of existing *nfxB*, *pilW*, and *kinB* mutations, appears to be responsible for the high-level resistance (Tables S1 and S2).

To isolate clones with high-level resistance and figure out mutations conferring high-level resistance in the low-dose regimen, the bacterial populations from the third cycle were plated on agar plates containing 20 mg/L fleroxacin and a portion of the lineages approximately ~3 × 10^−5^ of the cells exhibited markedly high-level resistance, with MIC values for fleroxacin exceeding 64 mg/L. The whole-genome sequencing results showed that one clone (Sequence Type 30) had mutations in *gyrA* (T83I), *nfxB* (frameshift), *pilW* (frameshift), and *kinB* (frameshift) while the other clone (Sequence Type 7) accumulated mutations in *gyrA* (D87Y), *mexR* (frameshift), and *kinB* (frameshift). We also pooled the isolated 31 clones together and subjected the pooled genomes to whole-genome sequencing to figure out the factions of different mutations. The mutation frequencies of *pilW* and *kinB* are nearly 100% percent, *mexR* and *gyrA* (D87Y) are 76%, *nfxB* and *gyrA* (T83I) are 17%, and *gyrB* (S466F) is 6.5% (Table S3). These results suggest that the evolution of high-level fleroxacin resistance under the low-dose or high-dose regimens both require combinational mutations of DNA gyrases and negative regulators of efflux pumps.

### Proteomic analysis of resistant populations evolved from low-dose and high-dose exposure modes

**Fig 3:**
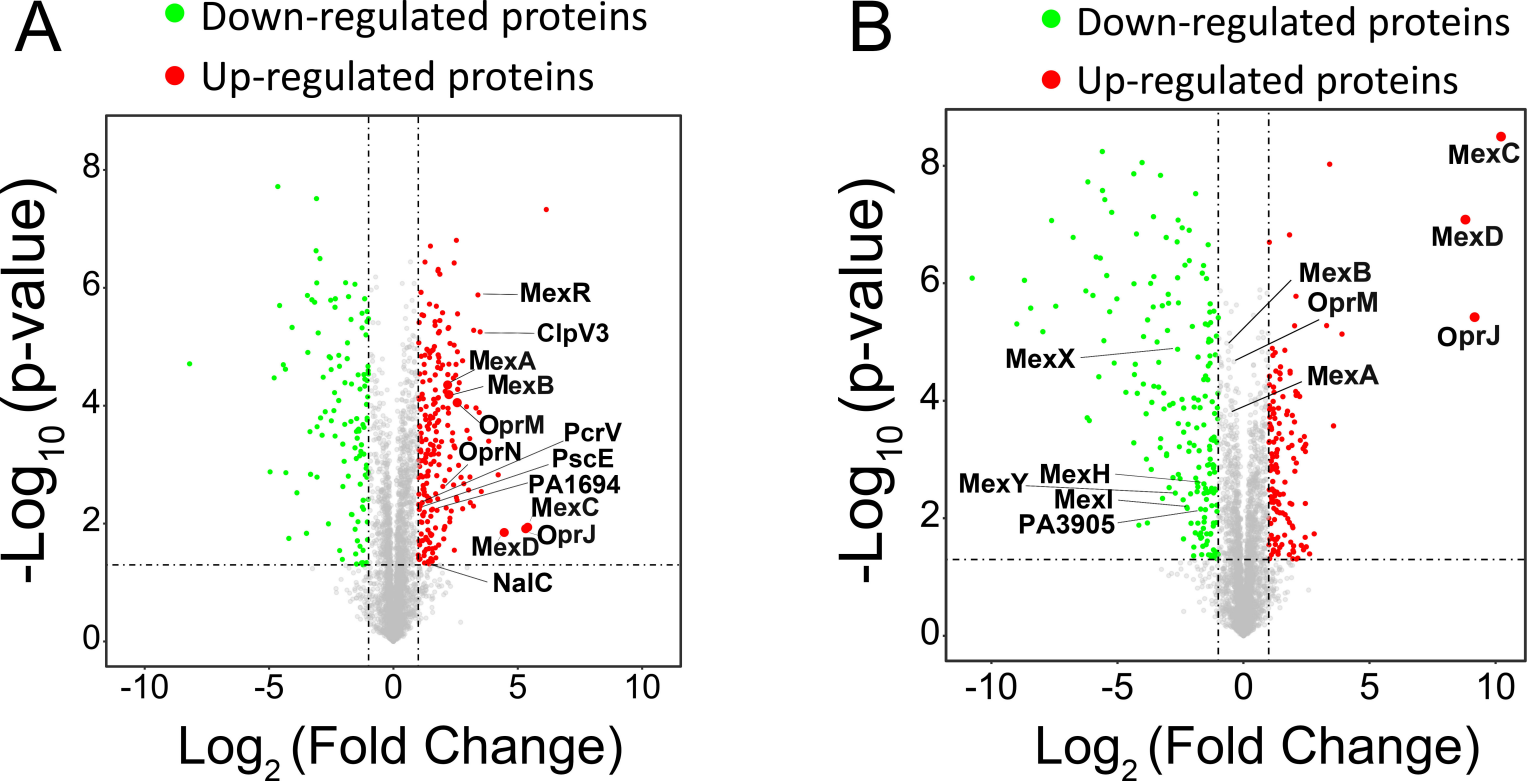
Proteomic analysis of resistant populations evolved from low-dose and high-dose exposure modes compared to the wild-type *P. aeruginosa* PAO1. Two volcano plots showed the differentially expressed proteins in the resistant strains evolved from low-dose exposure mode (**A**) and high-dose exposure mode (**B**) compared with the wild-type strain. For each protein, the −Log10 (*P* value) is plotted against its Log2 (fold change). Proteins up-regulated (*P* value < 0.05, fold change > 2) in the resistant strain are colored in red while proteins down-regulated (*P* value < 0.05, fold change < −2) are colored in green.

To explore how varying evolutionary pressures from low-dose and high-dose regimens shape protein expression profiles in *P. aeruginosa*, we employed quantitative proteomics to assess differentially-expressed proteins in end-point populations evolved from low-dose or high-dose fleroxacin exposure modes, comparing these with the ancestral wild-type populations. Z-score hierarchical clustering successfully grouped validated 241 protein samples from wild type to resistant strains evolved from low-dose or high-dose exposure mode (*P* value < 0.05), indicating the high reproducibility of our quantification (Fig. S3). Distinct responses were evident: 231 proteins were up-regulated and 120 down-regulated in low-dose-evolved populations, versus 146 up-regulated and 200 down-regulated in high-dose-evolved populations, using greater than twofold change and *P* value < 0.05 as criteria (Fig. 3A and B). The expressions of proteins like MexAB-OprM, MexR, NalC, OprN, H3-T6SS (ClpV3), T3SS (PA1694, PcrV, PscE) were up-regulated in the low-dose-evolved populations, contrasting with the high-dose-evolved population’s down-regulation of MexXY, MexHI, and several T6SS effectors (PA3905) (Tables S4 and S5). This differential protein expression suggests distinct adaptive strategies and potentially provides the genetic foundation of divergent resistance spectra in fleroxacin-resistant strains evolved from two antibiotic exposure modes (Fig. 1D).

Despite the pronounced differences in protein expression profiles, the populations evolved from low-dose and high-dose exposure modes still sharing 83 up-regulated and 80 down-regulated proteins, respectively, suggesting some degree of convergent evolution in response to low-dose and high-dose selection regimens. This convergence was particularly evident in the up-regulation of proteins associated with the efflux pump MexCD-OprJ and down-regulation of proteins involved in quorum sensing, biosynthesis of secondary metabolites like phenazine, type 6 secretion systems (T6SS). Remarkably, MexCD-OprJ production was elevated by over 1,000-fold in high-dose-evolved populations and 32-fold in low-dose-evolved populations, correlating with *nfxB* mutation frequencies (95% versus 20%) (Fig. 2B). This pump can extrude 4-hydroxy-2-heptylquinoline, a precursor of the *Pseudomonas* Quinolone Signal (PQS), resulting in diminished PQS levels influencing the expression of virulence factors and formation of biofilm (38). Combined with the fact that the MexAB-OprM efflux pump is only up-regulated in low-dose-evolved populations, our proteomic data results suggest that the divergent resistance spectra evolved under the two antibiotic exposure modes are likely to be caused by different expression levels in efflux pumps. With only increased production of MexAB-OprM and MexCD-OprJ detected in the proteomic analysis, these two efflux pumps appear to be more efficient at expelling fleroxacin than MexEF-OprN. This could be explained by the fact that MexEF-OprN has a more selective substrate profile, primarily expelling smaller, less hydrophobic molecules, and is, therefore, less effective at pumping out compounds like fleroxacin.

### High-dose fleroxacin selects the high-tolerance phenotype conferred by *nfxB* loss-of-function mutations

**Fig 4:**
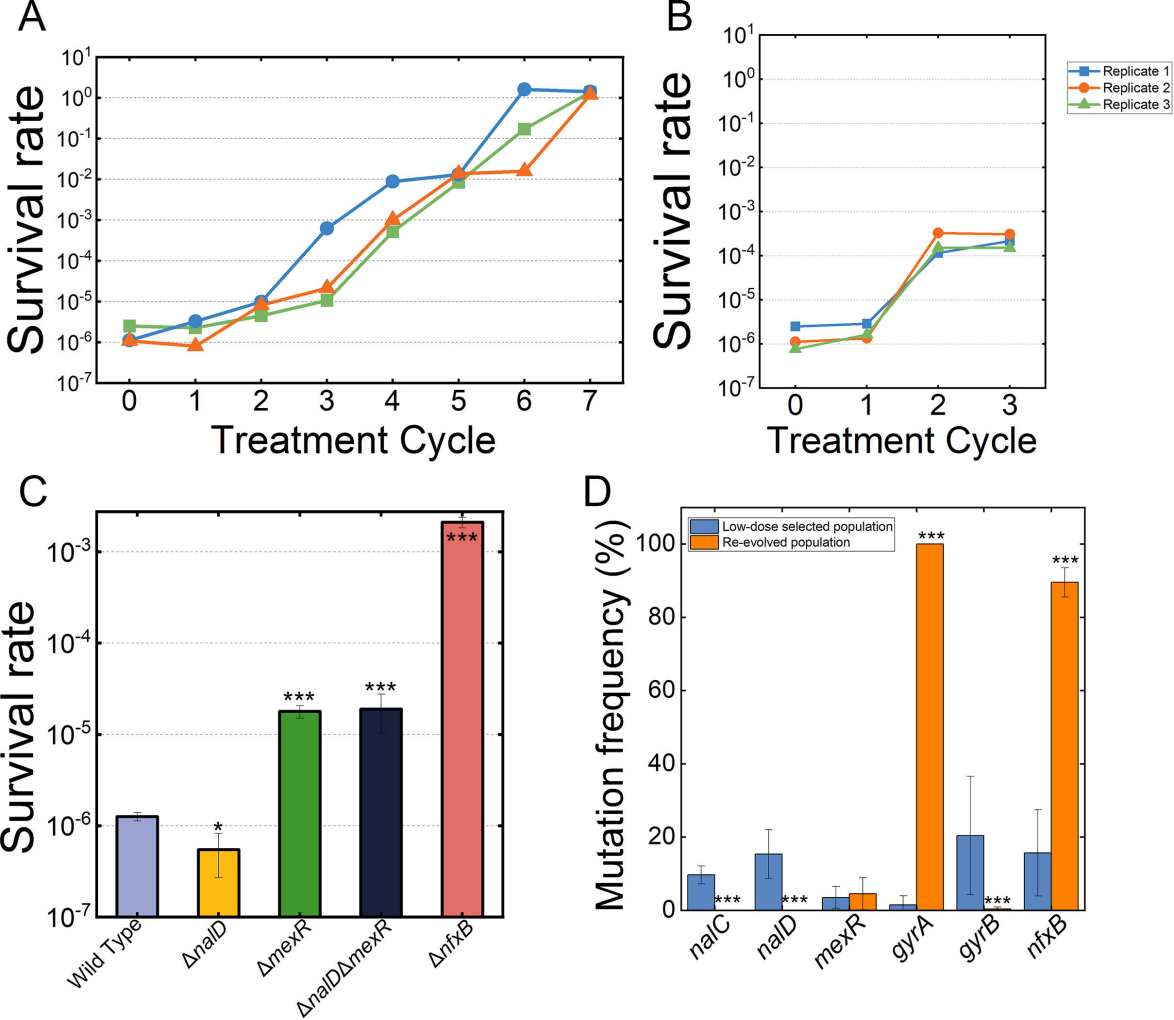
High-dose fleroxacin selects the high-tolerance phenotype conferred by *nfxB* loss-of-function mutations. (**A**) Tolerance was synchronously evolved and step by step in high-dose exposure mode. Survival rate of evolved strains in each cycle was measured. Three independent replicates were colored in blue, green, and orange, respectively. (**B**) Tolerance barely evolved in low-dose exposure mode. Survival rate of evolved strains in each cycle was measured. Three independent replicates were colored in blue, green, and orange, respectively. (**C**) Survival assays after 6 h in the wild-type *P. aeruginosa* PAO1 and its four different derivatives mentioned above. Three independent experiments were performed and the error bars were calculated. (**D**) High-dose exposure mode could select *nfxB* mutant. Blue histogram indicated the frequency of different genes in the resistant population (MIC = 32 mg/L) evolved from low-dose exposure mode. Orange histogram indicated the frequency of different genes in the resistant population re-evolved from high-dose exposure mode. Error bars denote standard deviation (*n* = 3). Student’s *t*-test was used, *P* < 0.05 was displayed as *, *P* < 0.01 was displayed as **, and *P* < 0.001 was displayed as ***.

Although both low-dose and high-dose treatment led to mutations of resistance-related genes including *nfxB*, *gyrA,* and *gyrB*, additional resistance-related mutations such as *nalC* and *nalD* only exist in the low-dose treated populations. It was previously demonstrated that lethal dose antibiotics often select high-tolerance mutants, which may occur in a higher probability and persist in lethal concentration of antibiotics for a much longer time, facilitating the evolution of high resistance (39). We, therefore, speculate that the selection outcome of the low-dose and high-dose regime may differ in antibiotic tolerance, with dose-dose treatment leading to high tolerance. To test this, we monitored the tolerance toward fleroxacin for low-dose and high-dose treated populations before high-resistant (>20 mg/L) mutants emerged, and found tolerance gradually increased for the high-dose treated populations after cycle 3, with the survival rate increased by at least 10^3^ in cycle 4 (Fig. 4A). For the low-dose treated populations, a slight increase was observed for the tolerance (Fig. 4B).

Our sequencing data indicate that the frequency of *nfxB* mutations reached more than 90% in cycle 5, whereas the frequency of *gyrA* and *gyrB* mutations is below 20%, suggesting that the high tolerance may be contributed by the *nfxB* mutations rather than the *gyrA*/*gyrB* mutations (Fig. 2D). Previous work indicated that enhanced efflux activity facilitates drug tolerance in bacterial persisters (40), we therefore constructed deletion mutants of *nfxB*, *mexR,* and *nalD*, which results in overexpression of MexCD-OprJ and MexAB-OprM, respectively. All these mutants showed similar growth rates (Fig. S4) and moderate increase of the MICs (twofold to fourfold) compared with the wild-type strain (Table 1). Notably, the survival rate of Δ*nfxB* strain is increased by 10^3^-fold, which is comparable to the fold-changes of the survival rate of evolved populations under high-dose treatment. In contrast, the survival rate of Δ*mexR* only increased by 10-fold, whereas Δ*nalD* showed a slightly decreased survival rate (Fig. 4C). These results indicated the *nfxB* loss-of-function mutation, which results in overexpression of the MexCD-OprJ efflux pump and strongly selected by high-dose fleroxacin treatment, conferred high antibiotic tolerance.

**TABLE 1:** MICs of wild-type *P. aeruginosa* PAO1 strain and its derivatives toward fleroxacin

	Wild type	Δ*nfxB*	Δ*mexR*	Δ*nalD*	Δ*nalD* Δ*mexR*	Δ*pilW*	Δ*kinB*	gyrA (H45Q)	gyrA (D72G)	gyrA (T83I)	gyrA (D87Y)	gyrB (S466F)	gyrB (P749S)
MIC (mg/L)	2	8	8	4	8	2	2	4	4	8	8	4	2

Based on the above findings, we reasoned that if the low-dose selected populations were subjected to high-dose treatment cycles, the mutations conferring high tolerance would be selected. To test this, we re-evolved the low-dose selected population using the high-dose treatment regime for seven cycles and re-sequenced the end-point populations. Consistent with our hypothesis, no mutations of *nalC*/*nalD* were detected in the re-evolved populations, and mutations of *mexR* were barely affected (from 3.4% to 4.5%). In contrast, in all three of the re-evolved populations, the frequency of *nfxB* mutation was more than 85%, further supporting the role of *nfxB* in antibiotic tolerance (Fig. 4D). It is interesting to note that all three of the re-evolved populations had mutation frequencies of 100% for *gyrA* (D87Y), suggesting re-evolved the low-dose selected population using the high-dose treatment regimen would drive the evolution of high-tolerance and high-resistance phenotype.

### Combination of target mutation and enhanced efflux pump expression leads to high-level fleroxacin resistance

**Fig 5:**
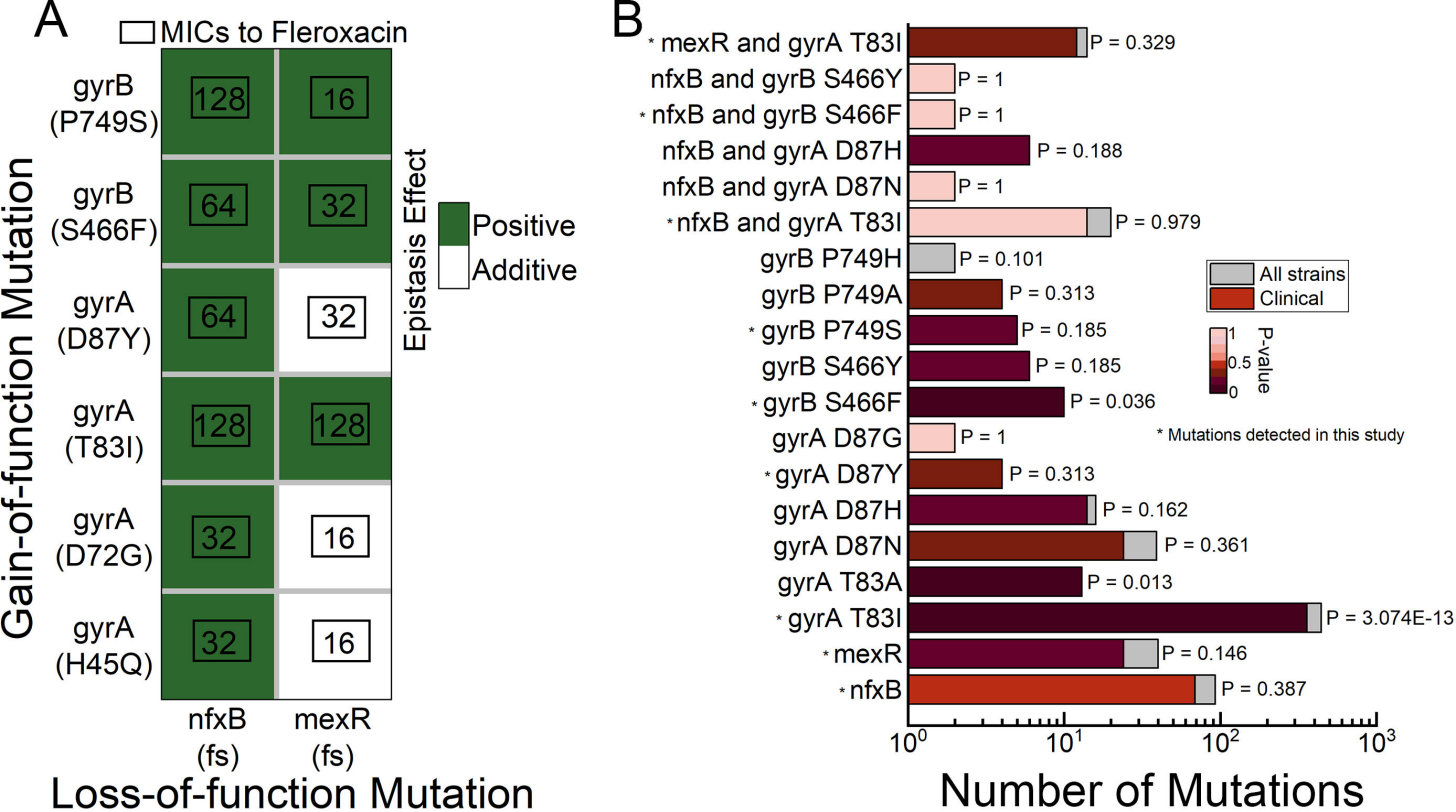
Combinations that lead to high-level fleroxacin resistance are prevalent in clinical and environmental strains. (**A**) Epistasis effect of *gyrA*/*gyrB* gain-of-function mutations and *nfxB*/*mexR* loss-of-function mutations. Positive effect and additive effect were colored with green and white, no negative effect was detected in this study. MICs of combinations of *gyrA*/*gyrB* mutations and *nfxB*/*mexR* mutations were shown in the black frame. (**B**) *nfxB*/*mexR*, *gyrA*/*gyrB* mutation, and their combinations are highly prevalent in clinical and environmental strains. Mutations in coding sequences were searched for in a database of 1,173 genomes downloaded from NCBI. Bars indicate the number of strains with the specific mutation from our data set (gray, total number; red, clinical strains). The significance and *P* values of the overrepresentation of each mutation within the subset of clinical strains are colored in varying degrees of red, from highly to not significant (dark to light red, respectively). Stars denote mutations tested in this study. The y-axis labels consist of gene names, nucleotide positions, and combinations, respectively.

We next examined how the putative resistance mutations contributed to the high level of resistance (>9.6 mg/L) and chose the classic resistance-related genes (*gyrA*, *gyrB*, *nfxB*, *mexR*) and genes with high-frequency (>80%) mutations (*kinB* and *pilW*) from our evolution experiments. For *nfxB*, *mexR*, *kinB,* and *pilW*, we constructed the in-frame deletion, as these four genes all have loss-of-function mutations in the evolved populations. For *gyrA* and *gyrB*, we expressed the mutant variants of each gene from a medium-copy plasmid (pRK415) without knocking out the corresponding chromosome genes. Mutations of *nfxB*, *mexR*, *gyrA,* and *gyrB* showed moderate (twofold to fourfold) increase of MIC, except for the *gyrB* (P749S)-expressing mutant (Table 1). Deletion of *kinB* or *pilW* did not impact the bacterial MIC, suggesting these two are growth-media adaptation mutations (Table 1). We then overexpressed the variants of *gyrA* or *gyrB* in the genetic background of Δ*nfxB* or Δ*mexR* strain, generating 12 combinations of mutations. All the mutants bearing these two-step mutations have MICs exceeding the original MPC value (9.6 mg/L), explaining the emergence of some of these combinations in our evolution experiment. Intriguingly, for the *gyrA* (T83I), *gyrA* (D87Y), and *gyrB* (P749S) expressing strains, deletion of *nfxB* results in 16-, 8-, and 64-fold increase in MIC, indicating a synergistic effect between these *gyrA* gain-of-functions mutations and *nfxB* loss-of-function mutation, i.e., the resistance of the double mutations exceeds the expectations from the additive effects of the individual mutations (41). The *mexR* deletion mutation also showed a synergistic effect with the *gyrA* (T83I) and the *gyrB* mutations (Fig. 5A). These results revealed that fluoroquinolone target mutations and increased efflux pump expression jointly conferred high resistance toward fluoroquinolone, with increased efflux pump expression synergistically potentiating effects of certain target mutations on fluoroquinolone resistance.

### Prevalence of *nfxB*/*mexR-gyrA*/*gyrB* mutation combinations in clinical and environmental strains

Since laboratory evolution protocols do not necessarily reflect the clinical environments, we next investigated the prevalence of the identified gene mutations in a library of 1,133 *Pseudomonas aeruginosa* strains with complete genome sequences from the NCBI database. Mutations in *gyrA* H45/D72/T83/D87 and *gyrB* S466/P749 were considered gain-of-function mutations whereas frame-shift mutations as well as mutations in the DNA-binding and dimerization region of *nfxB* and *mexR* were considered as potential loss-of-function mutations. We calculated the total abundance of each mutation and their combinations in strains classified as clinical (*n* = 773) and environmental or other (*n* = 360), respectively. Overall, 22 of the 41 mutations/mutation combinations were not identified in any of the 1,133 genomes, including *gyrA* H45Q, *gyrA* D72G, and *nfxB-gyrA* D87Y. The remaining 19 were present in at least 2, and up to 443, of the isolates. Only three of the thirteen *gyrA*/*gyrB* loci (*gyrA* T83I, *gyrA* T83A, and *gyrB* S466F) were statistically overrepresented in clinical isolates (*P* < 0.05, Fisher’s exact test) (Fig. 5B). However, although *gyrA* D87Y and *gyrA* D87N have also been reported to be highly associated with fluoroquinolone resistance, these two mutations are not significantly enriched in clinical isolates (*P* = 0.313 and *P* = 0.361, Fisher’s exact test; Fig. 5B) (42). This is consistent with the previous notion that the NCBI annotations of strains as clinical or environmental do not reflect their antibiotic exposure history and it was suggested that mutation prevalence alone can be an indicator of potential clinical impact (43). Notably, the *nfxB-gyrA* T83I and *mexR-gyrA* T83I mutations were abundantly present at levels similar to the *gyrA* mutations, highlighting the clinical significance of these two mutation combinations conferring high fluoroquinolone resistance.

### The efflux pump inhibitor PAβN prevents the evolution of high-level resistance in both laboratory and clinical strains

**Fig 6:**
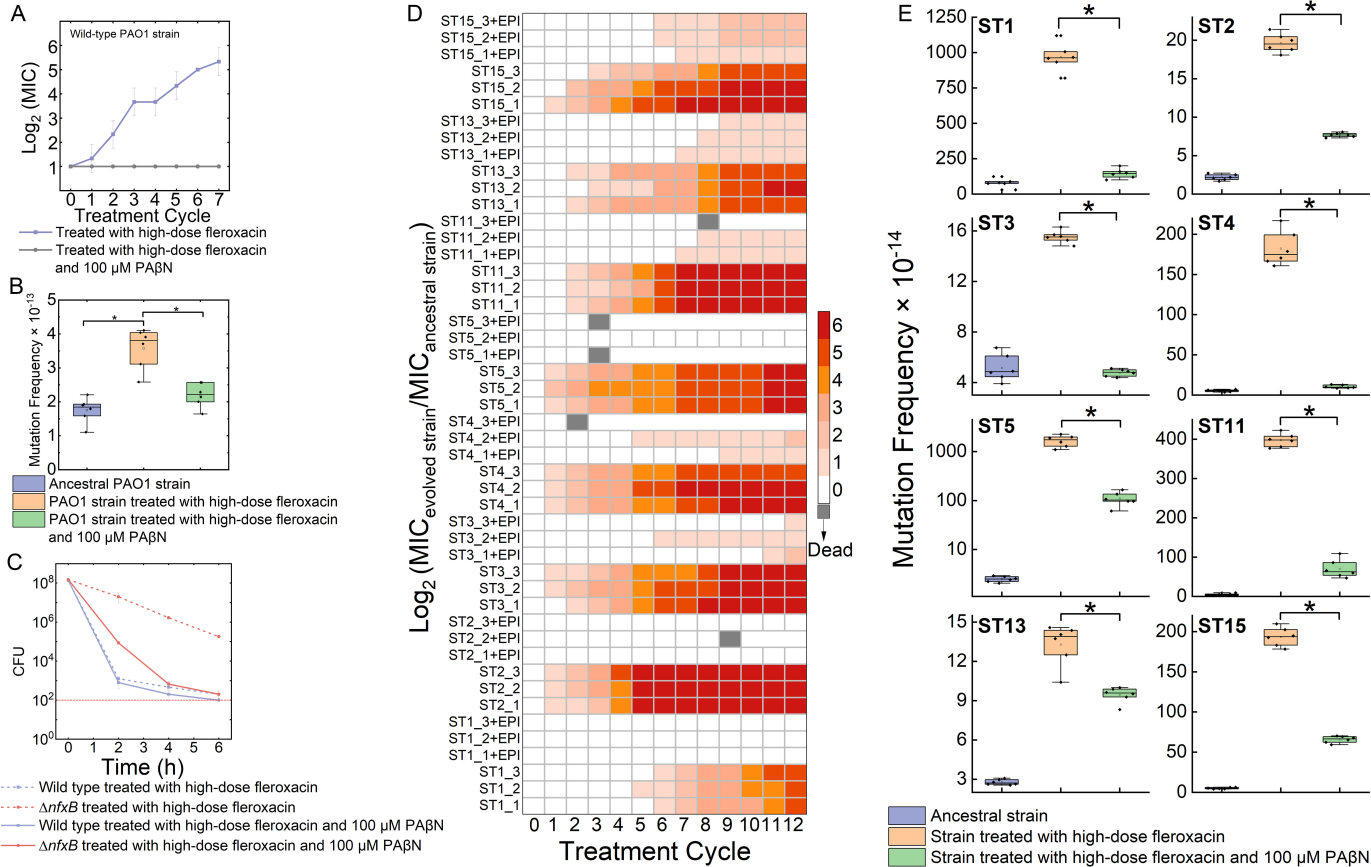
Efflux pump inhibitor PAβN could prevent evolution to high-level resistance in the high-dose exposure mode by decreasing the survival rate of efflux pump-overexpressed mutants. (**A**) A 100 µM PAβN could suppress the evolution to high-level resistance of wild-type *P. aeruginosa* PAO1. Three independent experiments were performed and the error bars were calculated. (**B**) Mutation frequency of ancestral wild-type PAO1 strain and evolved strain under one cycle of high-dose fleroxacin exposure mode with and without being treated with 100 µM PAβN. (**C**) Killing curve assays of 100 μM-PAβN-treated strains and control strains. (**D**) Resistance evolution of eight clinically isolated strains in high-dose fleroxacin exposure mode with and without being treated with 100 µM PAβN. MIC of each batch in different cycles was measured and the relative change of MIC to that of ancestral strains was displayed in the heatmap. (**E**) Mutation frequency of ancestral clinically isolated *P. aeruginosa* strain and evolved strain under one cycle of high-dose fleroxacin exposure mode with and without being treated with 100 µM PAβN.

As our data indicate that overexpression of efflux pumps like MexAB-OprM and MexCD-OprJ is mandatory to the evolution of high-level resistance, we postulated that by using effective efflux pump inhibitors, we could potentially prevent the evolution toward high-level resistance. We tested two efflux pump inhibitors, namely PAβN and 1-(1-naphthylmethyl)-piperazine (Fig. S1B and C) (44–47). Only PAβN could act as the genuine efflux pump inhibitor in *P. aeruginosa*, as indicated by its ability to enhance the antibiotic activity of fleroxacin (Fig. S5). We then conducted the high-dose fleroxacin treatment regimen in the presence of 100 µM PAβN. Bacterial populations only treated with fleroxacin quickly developed high fleroxacin resistance, while populations treated with fleroxacin combining PAβN maintained sensitivity within 70 generations (Fig. 6A). As it was recently revealed that efflux pump inhibitors decreased evolvability to antibiotic resistance in *E. coli* (48), we tested if it is also true in *P. aeruginosa*. We found PAβN could indeed reduce the evolvability to antibiotic resistance by showing PAβN suppressed the bacterial mutation frequencies under the high-dose fleroxacin treatment (Fig. 6B). In addition, PAβN significantly suppressed the fleroxacin tolerance of the *nfxB* mutant (Fig. 6C). Overall, these results demonstrate that the efflux pump inhibitor PAβN could prevent high-level resistance evolution by suppressing the resistance evolvability and antibiotic tolerance.

We next collected eight clinically isolated *P. aeruginosa* strains (ST1, ST2, ST3, ST4, ST5, ST11, ST13, and ST15) and tested the effect of PAβN on their resistance evolution. All these strains have an initial MIC of 2 mg/L. Under the high-dose fleroxacin exposure mode, populations of all the strains quickly developed high-level fleroxacin resistance. In contrast, PAβN suppressed resistance evolution (Fig. 6D) and evolvability (Fig. 6E) in the evolved populations. Taken together, our results suggest that the use of efflux pump inhibitors offers a new avenue to prevent the evolution of antibiotic resistance in clinical settings.

## DISCUSSION

The increasing challenge of multidrug resistance (MDR) is compounded by the scarcity of new antibiotic development (49). Not only is the development of novel antibiotics crucial in combating MDR, but it is also important to innovate antimicrobial therapies that prevent the evolution of resistance (50–54). In this work, we used experimental evolution and whole-genome sequencing to investigate the multi-step evolution mechanisms of fluoroquinolone resistance in *Pseudomonas aeruginosa*, an opportunistic pathogen notorious for its capacity to develop multi-drug resistance. We found that the loss-of-function mutation in either *nfxB* or *mexR*, which can result in the overexpression of efflux pump MexCD-OprJ or MexAB-OprM, is a critical step toward evolving high-level resistance to fleroxacin. The bioinformatic analysis also revealed a high prevalence of potential loss-of-function mutations in *nfxB* and *mexR* among both clinical and environmental *P. aeruginosa* isolates, highlighting the importance of efflux pumps in high-level resistance evolution. Our study highlights the importance of lab-based evolution experiments in deepening our understanding of antibiotic resistance mechanisms.

How to prevent the occurrence and propagation of multi-step, high-resistance mutants is essential in treating and managing drug-resistant pathogens. It was reported that by targeting a common mutation L28R of dihydrofolate reductase (DHFR) with a trimethoprim (TMP) derivative (4′-DTMP), the evolution of high TMP resistance in *E. coli* could be blocked, for the L28R mutation of DHFR has positive epistatic interactions with other resistance-conferring DHFR mutations (54). Here, we demonstrated that in *P. aeruginosa*, the combinations of mutations in the negative regulators of efflux pumps (*nfxB*/*mexR*) and DNA gyrase (*gyrA* H45, D72, T83, D87 mutation or *gyrB* S466, P749 mutation) contributed to the high fluoroquinolone resistance. By inhibiting the efflux pumps using PAβN, the high-level resistance evolution was prevented in clinically isolated *P. aeruginosa* strains, indicating efflux pumps are promising targets for preventing the high-level resistance evolution.

As a broad-spectrum efflux pump inhibitor, PAβN has been shown to be synergistic with multiple antibiotics, including macrolides, neomycin, β-lactams, and fluoroquinolones, highlighting its potential as an effective adjuvant therapy to combat antimicrobial resistance. The main mechanisms underlying its efficacy include acting as a competitive substrate for efflux pumps and inducing outer membrane permeabilization (44, 55–58). Moreover, the combination of azithromycin and PAβN could reduce QS-dependent virulence factor production in *P. aeruginosa* (59). Its combination with antibiotics could play a key role in overcoming efflux-mediated resistance in bacterial infections, but understanding its full pharmacokinetic and toxicological profile is essential for optimizing its use in clinical settings (60). The safety and synergistic effects of PAβN with neomycin were previously evaluated *in vivo*. The results showed that at a safe dose of 40 µg/g body weight, PAβN acted as a therapeutic adjutant of neomycin in ducks (56). Unfortunately, high levels of renal toxicity associated with efflux pump inhibitors (EPIs) have been observed (61–63). Although modifications to the PAβN structure have reduced its acute toxicity to a tolerable level, the presence of two cationic groups leads to prolonged tissue accumulation, presumably in acidic vesicles, which limits the feasibility of repeated dosing (64). Furthermore, the lack of preclinical and clinical data presents another significant hurdle for EPIs to become viable therapeutic options (65).

In conclusion, our research established that overexpression of efflux pump MexCD-OprJ or MexAB-OprM is a mandatory step toward evolving high-level resistance to fleroxacin. We demonstrated the potential of targeting efflux pumps to prevent the emergence of high-resistant mutants. Our findings suggest that developing potent inhibitors of these efflux pumps represents a promising approach to control the evolution of antibiotic resistance in chronic infections.

## MATERIALS AND METHODS

### Experimental evolution of resistance

In this study, we performed two regimens to make *Pseudomonas aeruginosa* evolve fluoroquinolone resistance under exposure to fleroxacin: (i) high-dose exposure mode: an overnight culture was diluted 1:100 into 5 mL Luria-Bertani (LB) medium supplemented with high concentration (20 mg/L) (~MIC × 10) of fleroxacin and incubated at 37°C for 6 h with shaking at 220 rpm. A volume of 2 mL of treated cultures was collected by centrifugation at 8,000 rpm for 5 min and washed three times with fresh LB medium. Collected cultures were resuspended and then regrown in a 5 mL fresh medium containing a subinhibitory concentration (0.2 mg/L) (~MIC × 0.1) of fleroxacin overnight till 1.5 OD_600_. This process was referred to as one cycle. A 1 mL of the regrown culture was used for analysis of each cycle and was frozen (39). (ii) Low-dose exposure mode: an overnight culture was diluted 1:100 into a fresh LB medium containing a sub-MIC concentration (1 mg/L) (~MIC × 0.5) of fleroxacin and incubated overnight. This process involved continuous exposure to antibiotics, and we also called it one cycle. At least three cycles were performed in each antibiotic exposure mode.

### Whole-genome sequencing and data analysis

Genomic DNA (gDNA) was extracted from 1 mL of overnight cultures of the ancestral strain and bacterial populations evolved from high-dose or low-dose exposure mode using the TIANamp Bacterial DNA Kit (TIANGEN BIOTECH CO., LTD., Beijing, China), according to the manufacturer’s instructions. NanoDrop spectrophotometer (Thermo Scientiﬁc, Waltham, MA, USA) accurately assayed the quality of gDNA, while 1% (wt/vol) agarose gel electrophoresis facilitated visual assessment. The gDNA was sent to the Novogene Co., Ltd. (Beijing, China) for whole-genome sequencing using the Illumina Nova6000 PE150 sequencing system (Illumina Inc., San Diego, CA, USA) with 200× coverage. Data quality control was performed as below: low-quality data including reads containing adapter contamination, low-quality nucleotides, and unrecognizable nucleotides (N) was removed. Bwa-mem2 was used to build a database and generate alignment with the reference genome of *Pseudomonas aeruginosa* PAO1 (GenBank accession number NC_002516.2) by using default settings (66). Samtools were used to call mutations with single nucleotide polymorphisms (SNPs) (67). Picard Tools were used to remove duplicated reads with default settings (68). Samtools was employed for variant filtering, with probabilistic realignment deactivated to mitigate false SNPs due to misalignments (67). Pindel was used to call mutations with insertions/deletions (69). The output variant call format file was generated by Bcftools (69, 70). Compared to the original NCBI reference PAO1 strain, the ancestral PAO1 strain used in this study already has no genomic differences.

### MIC assay

The MIC assay was conducted with moderate modifications based on previous studies (9). Wild-type *Pseudomonas aeruginosa* PAO1 and its mutant strains in the logarithmic growth phase (OD_600_ =  0.8) were diluted to a final concentration of approximately 10^5^ cells/well in LB medium and then inoculated into 96-well cell culture plates containing gradient concentrations (0.5–256 mg/L) of antibiotics. After incubation at 37°C for 20 h, the absorptions of each well were measured using a microplate reader at a wavelength of 600 nm (Absorbance Reader ELx808, BioTek) and MIC was the lowest concentration that inhibits visible growth.

### Survival assay

Survival assay was performed as previously reported with minor modifications (71). An overnight culture was diluted, and ~10^11^ cells were inoculated into a 5 mL LB medium supplemented with 20 mg/L fleroxacin for 6 h to kill sensitive cells. A 100 µL of culture was then placed on LB agar to calculate the CFUs after incubation at 37°C for 24 h and survival rates were calculated. Three biological replicate experiments were conducted and error bars were calculated.

### Construction of mutation strains

In-frame deletion mutants were constructed by two-step recombination (72). Flanking regions of ~1 kb of the target gene were amplified with primers listed in the supplementary table and ligated into the pk18mobsacB suicide vector. The constructed plasmid was transposed into *E. coli* WM3064, subsequently incorporated into the host strain via conjugation. The mutant strain was selected with 10% sucrose and confirmed by PCR. The strains with complementation of *gyrA*/*gyrB* in the genetic background of wild-type PAO1, Δ*nfxB,* and Δ*mexR* were performed as follows. The amplified *gyrA* or *gyrB* was ligated into the vector pRK415 (73). The constructed pRK415-*gyrA* or pRK415-*gyrB* was transposed into *E. coli* WM3064, subsequently incorporated into the host strain via conjugation. The resulting mutants were plated on the LB agar with 50 mg/L tetracycline in the wild-type background or 200 mg/L tetracycline in the Δ*nfxB* background and Δ*mexR* background. Primers used in this study have been listed in Table S6.

### Total protein extraction, digestion, and mass spectrometry analysis

Bacterial populations evolved from high-dose or low-dose exposure mode and wild-type *P. aeruginosa* were cultured in LB medium at 1.0 OD_600_ and collected. Samples underwent cellular lysis, after which proteins were thermally denatured (95°C/30 min) and then sonicated for 1 h (74). Mixing was subsequently dilated 10-fold in a solution comprising 25 mM Tris-HCl (pH 8.5) and 10% acetonitrile (ACN), followed by tryptic digestion at 37°C for an entire night. After digestion, total protein underwent 5% TFA acidification before injecting onto a pretreated, methanol and 0.2% acetic acid-equilibrated C18 column. The total peptide was washed three times with 0.1% acetic acid, eluted with 80% ACN and 0.1% acetic acid, and finally dried. For MS analysis, total peptides were dissolved in 0.1% FA and analyzed by Orbitrap Fusion Lumos mass spectrometer (Thermo Scientific). Raw data were processed with the DIA-NN software package against *P. aeruginosa* PAO1 sequence data. N-terminal protein acetylation and methionine oxidation served as variant modifications, while carbamidomethyl was fixed. Bioinformatics and statistics were conducted using Perseus software (75). Student *t*-test was used to determine the significance of differential protein expression between wild type and resistant bacterial cells, while Fisher’s exact test conducted function enrichment analysis. Statistical analysis utilized a *P* value ≤ 0.05 with a threshold of ≥1.5-fold change.

### Bioinformatic analysis

A total of 1,133 *Pseudomonas aeruginosa* strains with complete genome sequences were downloaded from the NCBI database. Mutations were detected with principles as below: for *nfxB* and *mexR*, mutations in the DNA-binding and dimerization region as well as frameshift were defined as potential loss-of-function mutations, as V143-G180, R21-F43 of *nfxB*, as well as L9-F17, D64-E76, E127-C138 of *mexR* (76, 77). Each mutation variant of *gyrA*/*gyrB* was found in the genome and defined as potential gain-of-function mutations. The software Diamond (v2.0.15.153) was used to generate alignment with the reference genome of *Pseudomonas aeruginosa* PAO1 (GenBank accession number NC_002516) by using settings with identity >50%, coverage >80%, E-value = 1E-5. We calculated the total abundance of each mutation and their combinations in strains classified as clinical (*n* = 773) and environmental or other (*n* = 360), respectively. Fisher’s exact test was used to determine expected differences in frequency for mutation frequency as is the standard in the field. All newly generated code and software essential for replicating the main findings of this study have been uploaded to github (https://github.com/cuiyinguniverse/Pseudomonas_aeruginosa_resistance).

### Mutation frequency assay

Mutation frequency (MF) assay was performed as reported previously (48). MFs were calculated by plating 1 mL (adjusted to ∼OD_600_ = 1) culture on 4 mg/L fleroxacin hard agar plates and incubated at 37°C for 48 h. The resultant colonies were counted and MFs were calculated as resistant colonies/CFU.

## Data Availability

DNA sequencing data are available in NCBI SRA with accession number PRJNA998700. The MS raw files and proteome sequences of *P. aeruginosa* PAO1 have been deposited to the ProteomeXchange Consortium via the PRIDE partner repository with the data set identifier PXD044054 (78, 79).

## SUPPLEMENTAL MATERIAL

Supplemental figures spectrum.02981-24-s0001.pdf Fig. S1 to S5.

Table S1 spectrum.02981-24-s0002.xlsx Mutations with frequency > 10% detected in the low-dose exposure mode in different treatment cycles.

Table S2 spectrum.02981-24-s0003.xlsx Mutations with frequency > 10% detected in the high-dose exposure mode in different treatment cycles.

Table S3 spectrum.02981-24-s0004.xlsx Mutations with frequency > 10% in the clones with high-level resistance.

Table S4 spectrum.02981-24-s0005.xlsx Down-regulated and up-regulated proteins in the resistant population evolved from low-dose exposure mode compared to wild-type *P. aeruginosa* PAO1 strain.

Table S5 spectrum.02981-24-s0006.xlsx Down-regulated and up-regulated proteins in the resistant population evolved from high-dose exposure mode compared to wild-type *P. aeruginosa* PAO1 strain.

Table S6 spectrum.02981-24-s0007.xlsx Primers used in this study.

ASM does not own the copyrights to Supplemental Material that may be linked to, or accessed through, an article. The authors have granted ASM a non-exclusive, world-wide license to publish the Supplemental Material files. Please contact the corresponding author directly for reuse.
