# Supplementary material for: Targeting efflux pumps prevents the multi-step evolution of high-level resistance to fluoroquinolone in Pseudomonas aeruginosa
Source: Microbiol Spectr. 2025 Feb 21;13(4):e02981-24. doi: 10.1128/spectrum.02981-24 (PMC11960432; doi:10.1128/spectrum.02981-24)
Supplement: Supplemental figures — Fig. S1 to S5. [file spectrum.02981-24-s0001.pdf]

Supplementary Information

Targeting efflux pumps prevents the multi-step evolution of high-level resistance to fluoroquinolone in *Pseudomonas aeruginosa*

Xiao-Quan Yu[a], Hao Yang[b], Han-Zhong Feng[b], Jun Hou[b], Jun-Qiang Tian[a], Shao-Min Niu[a], Chong-Ge You[c], Xuan-Yu Tao[d], Si-Ping Zhang[b], Zhi-Ping Wang[a*] and Yong-Xing He[b*]

Table of Contents

Figures

Figure S1.--3

Figure S2.--4

Figure S3.--5

Figure S4.--6

Figure S5.--7

Author Contributions ---8

Corresponding Authors ---8

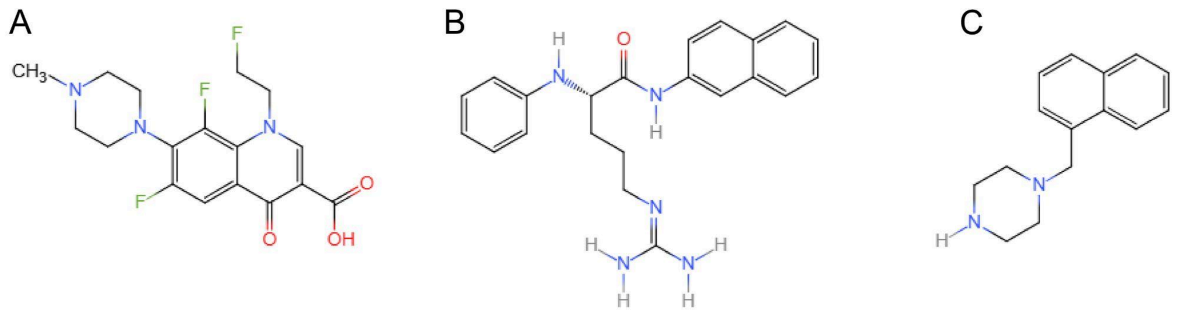

Figure S1. Structures of fleroxacin (A), phenylalanine-arginine β-naphthylamide (PAβN) (B) and 1-(1-naphthylmethyl)-piperazine (NMP) (C).

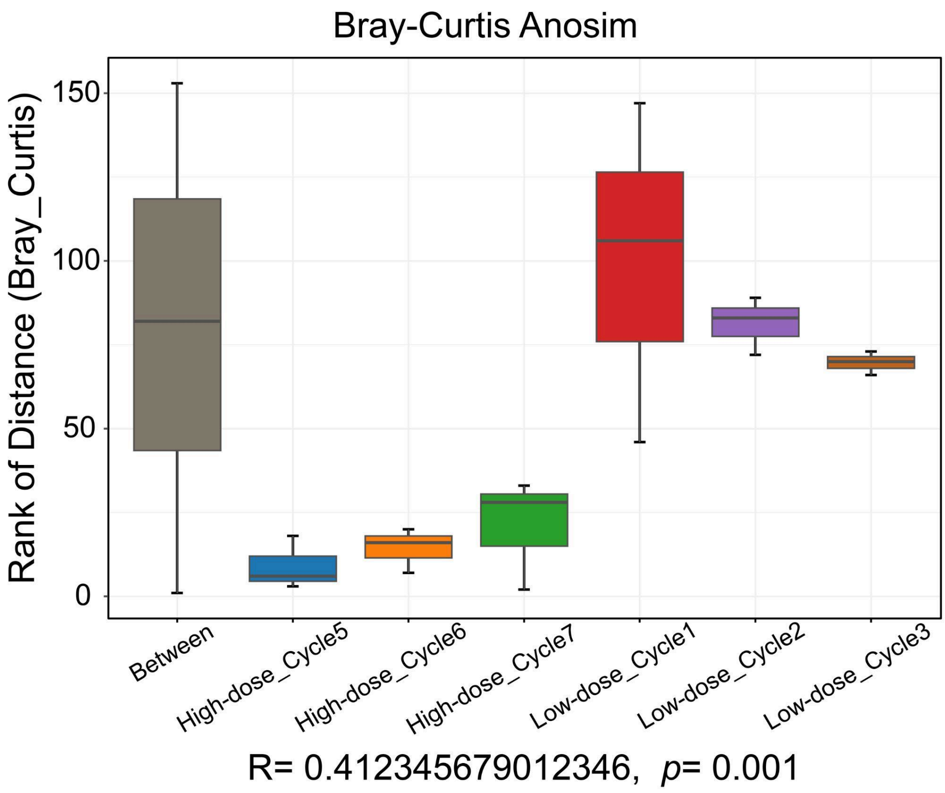

Figure S2. ANOSIM analysis based on Bray-Curtis algorithm used to test whether the difference between populations is more significant than the difference within populations. Different colors represent different populations.

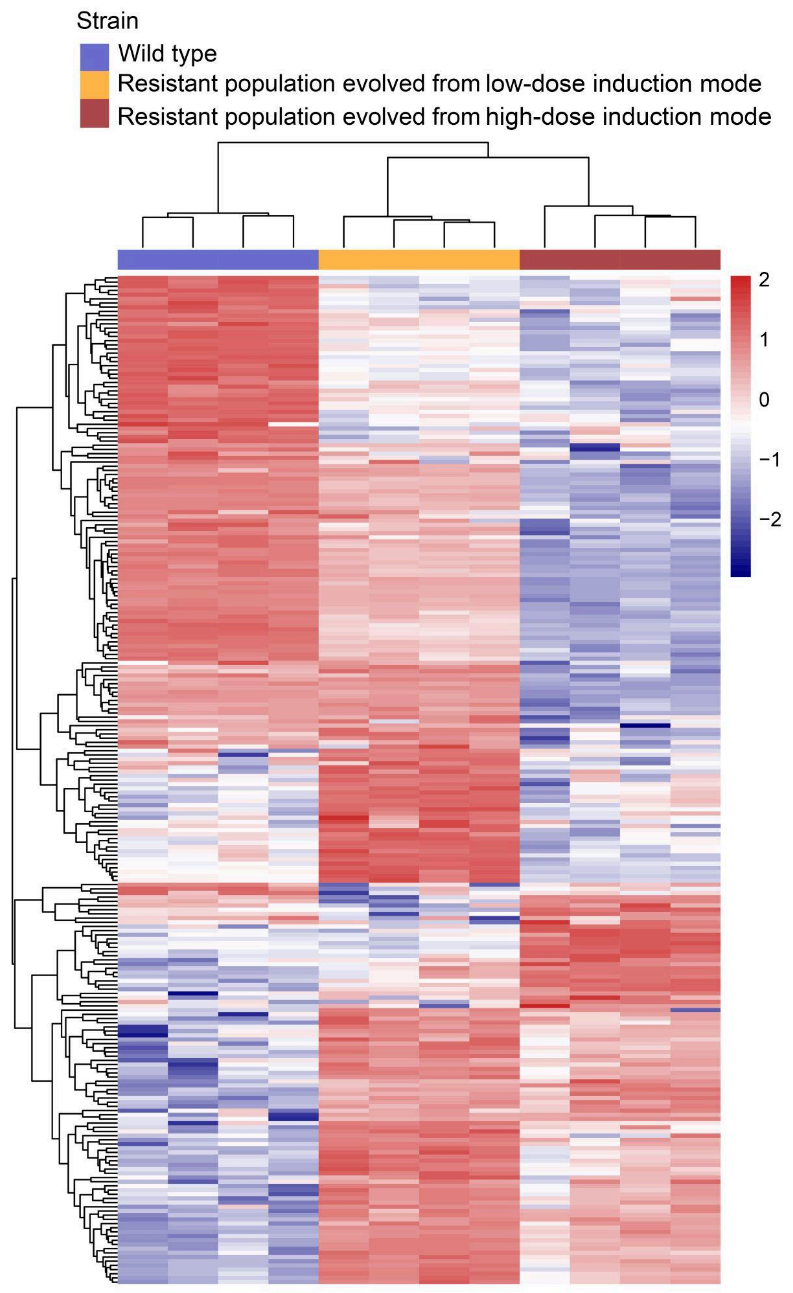

Figure S3. Hierarchical clustering based on an analysis of variance (ANOVA) test (FDR 5%) to evaluate the reproducibility of the proteome quantification between the resistant strains evolved from low-dose or high-dose induction mode based on *P. aeruginosa* PAO1 wild type, respectively.

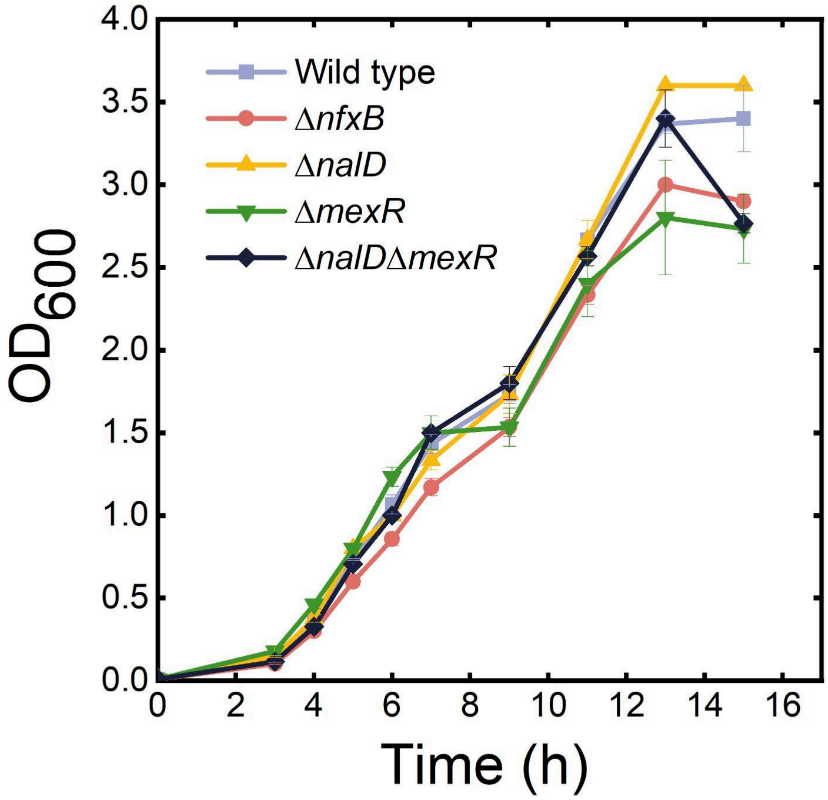

Figure S4. Wild-type *P. aeruginosa* PAO1 and its derivatives have similar growth rates.

Growth curve assays in the five different strains mentioned above. Three independent replicates were performed and error bars represent standard error of mean ($n = 3$).

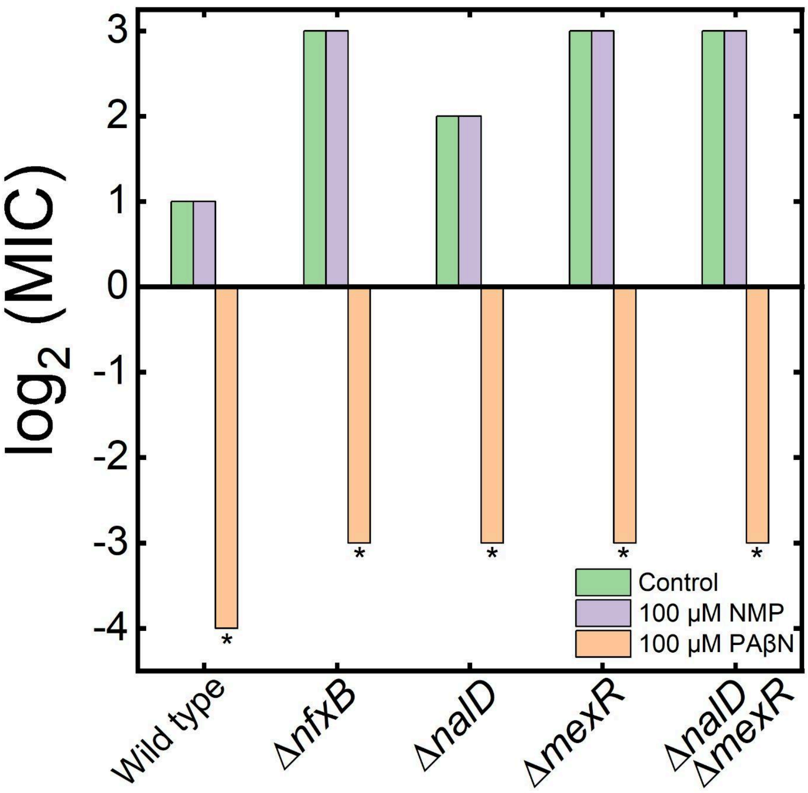

Figure S5. 100 μM PAβN increases susceptibility to fleroxacin of wild-type *P. aeruginosa* PAO1and its derivatives, but NMP does not.

MIC assays in wild-type *P. aeruginosa* PAO1and its derivatives to fleroxacin with 100 μM PAβN and 100 μM NMP. Three independent experiments were performed and the error bars were calculated. By using Student's t test, *P*-values were calculated, $P < 0.05$ is displayed as *.

Author Contributions

Y.X.H., Z.P.W. and X.Q.Y. conceived of the study and designed experiments. X.Q.Y carried out experiments. H.Y., H.Z.F and J.H. carried out bioinformatic work and data analysis. X.Q.Y and Y.X.H. wrote the paper, which was edited by all authors.

Corresponding authors

Correspondence to Zhi-Ping Wang and Yong-Xing He.
